# Telemedicine in the Western Cape Department of Health during the first peak of the COVID-19 pandemic: Leveraging data to save lives by activating a telemedicine response

**DOI:** 10.4102/phcfm.v13i1.2954

**Published:** 2021-05-20

**Authors:** Neal J. David, Zameer Brey, Muzzammil Ismail

**Affiliations:** 1 Division of Family Medicine and Integrated Palliative Care, Faculty of Health Sciences, University of Cape Town, Cape Town, South Africa; 2 Department of Health, Metro Health Services, Western Cape Government, Cape Town, South Africa; 3 Bill and Melinda Gates Foundation (BMGF), Seattle, United States of America; 4 Bill and Melinda Gates Foundation (BMGF), Cape Town, South Africa; 5 Department of Health, Health Impact Assessment (HIA), Western Cape Government Health (WCGH), Cape Town, South Africa; 6 School of Public Health and Family Medicine, Faculty of Health Sciences, University of Cape Town, Cape Town, South Africa

**Keywords:** data analysis, risk-stratification, telemedicine, coordination of care, diabetes, patient-centric

## Abstract

The pandemic caused by coronavirus disease 2019 (COVID-19) has put health systems across the globe under strain. There has been much suffering and loss, but a silver lining is emerging – a growing list of deeply contextualised, resource-light and patient-centric innovations that are showing the promise of reshaping health care delivery as we know it. Some of these innovations were lying latent in the system, waiting for the ‘dots to be joined’. The Western Cape was the first province in South Africa to experience a COVID-19 wave from May 2020 to July 2020, with 60–70 deaths being reported daily. To bend the mortality curve during this crisis was not easy but was made possible using a rudimentary telehealth system. This project represents an exemplar of innovation, built out of necessity to save lives and may well become a staple component of the health service in a post-crisis era.

## Background

When the first wave of the coronavirus disease 2019 (COVID-19) global pandemic hit the shores of the Western Cape, it became evident that many of the poorest outcomes, namely severe illness, hospitalisations and deaths, were affecting people with pre-existing diabetes. Our data analysis relating to COVID-19 mortality showed that 52% of deaths were amongst diabetic patients.^1^ Further data analysis indicated that advanced age and the presence of chronic kidney disease (CKD) in diabetic patients placed them at highest risk within the total cohort, with an in-hospital mortality rate in excess of 60%. However, earlier admission and improved glycaemic control showed a trend of lower mortality.^2^

An initiative led by a Family Physician and a Public Health Registrar was developed to address this health care crisis and to coordinate an operational response. Two basic principles of Family Medicine were applied in the development of this response. One is the enhancement of health and prevention of disease and the other is coordination of care by a health care team. In this context, application of the former required us to identify these high-risk patients before they decompensated and offer them an intervention. Application of the latter required us to achieve consensus amongst the various stakeholders who would be required to participate, either directly by providing a service or indirectly by means of support.

## Developing the intervention

With the understanding that diabetes mellitus, advanced age and CKD were the most sinister risk factors with COVID-19, a series of engagements with potential stakeholders were held. These included front-line clinicians, Emergency Medicine specialists, Public Health specialists, Family Physicians, Endocrinologists, Virologists and high-level managers in the Department who supported and guided the implementation of the programme. The result was a shared recognition of the risk factors that had been identified and the aim of offering earlier admission and acute glycaemic control of high-risk patients.

With a clearly articulated problem statement and key system capabilities (as listed below), we determined that a virtual intervention using a telemedicine approach was the natural next step. Based on available data and system-wide engagements, a risk-stratified algorithm was developed that could immediately classify patients according to their risk as soon as they were diagnosed with COVID-19. The three categories were defined as follows:

- High Risk
  ■ All COVID-19 diabetics > 60 years old or COVID-19 diabetics of any age with CKD
- Moderate Risk
  ■ All COVID-19 diabetics < 60 years old with any other additional comorbidity (except CKD)
- Low Risk
  ■ All COVID-19 diabetics < 60 years old with no other comorbidity

**FIGURE 1:**
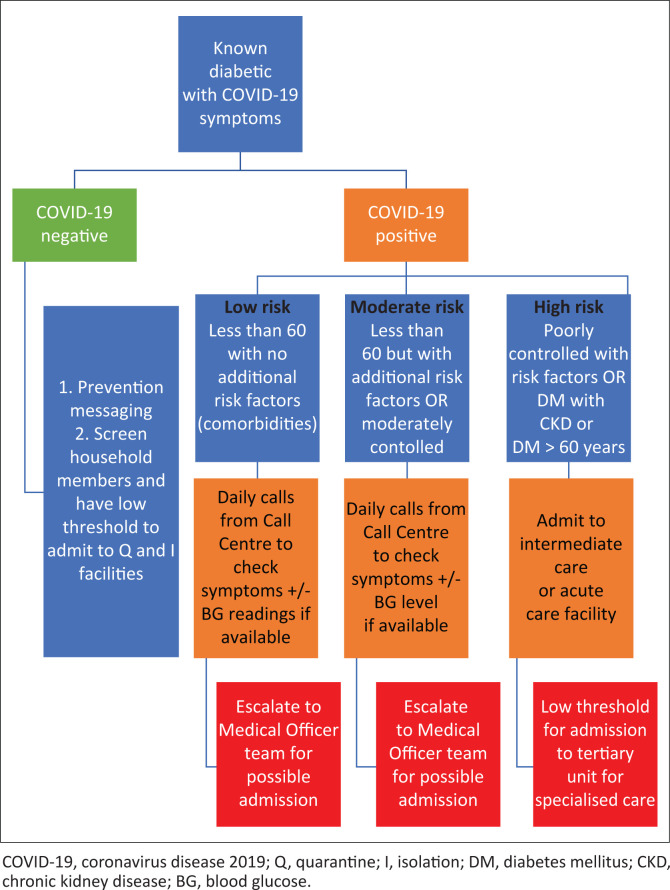
Risk stratified approach to COVID-19 diabetics.

Importantly, the algorithm excluded patients who were already deceased or admitted. Each risk stratum had a clear escalation pathway linked with it. All high-risk patients would be called by a Medical Officer (MO) and offered immediate pre-emptive admission to intermediate care facilities or remote monitoring by daily follow-up calls from the MO until 10 days post-diagnosis if they declined the offer of admission. Acutely ill patients would be admitted to acute care facilities as emergencies. Low- and moderate-risk patients would receive daily follow-up calls by Call Centre Agents following a script with escalation to the MO team if any red flags were observed (Figure 1).

It is important to identify the key inputs that made it possible to develop this intervention. The Western Cape Department of Health (WCDoH) has invested in building its own health information exchange for almost a decade. The Provincial Health Data Centre (PHDC) links almost two dozen sources of data across institutions in the province with a view to creating a rich and longitudinal picture of an individual patient’s engagement with the system over time. The system was in a strong position when COVID-19 emerged and became the default information system for management and operational purposes. For this intervention it was able to provide all the relevant contact details and public sector health interface data for the three risk strata with COVID-19 in almost real time.

On the health system side, there were several clinicians who were too high-risk to work on the frontlines (i.e. pregnant, with comorbid disease, etc.), but who were keen to be involved in the COVID-19 response. Finally, there was some spare capacity in the field hospital settings to accommodate a few patients during the first wave, which enabled referrals and early admission when required.

This meant that we had a running list of newly diagnosed COVID-19 positive, high-risk patients and we had an existing set of resources allowing us to follow these patients remotely and to facilitate early admissions. With no new telephony equipment, this combination of circumstances – existing high-quality data, human resources and bed availability – allowed us to develop this ultra-basic, but high impact telehealth system within a 3-week period. Telehealth services have emerged triumphantly across many geographies during the pandemic and have been shown to improve health care services.^3^

Code-named VECTOR (Virtual Emergency Care Tactical OpeRation), was initially operationalised with a group of six MOs who became active once they understood the data case supporting the intervention and were familiar with the Standard Operating Procedure. Every morning, data from the PHDC would run through the algorithm to generate a line list of high-risk diabetic patients with a COVID-19 diagnosis in a 10-day window, and these patients would be allocated to the MOs to call using the high-risk approach described above. Once the large backlog of high-risk patients was cleared, the service was extended to medium- and low-risk patients, initially in the Cape Town Metro and subsequently in rural districts of the Western Cape. Within two months the service reached all COVID-19 diabetic patients with a public sector health record in the province, even if their COVID-19 diagnosis was made in a private laboratory.

**FIGURE 2:**
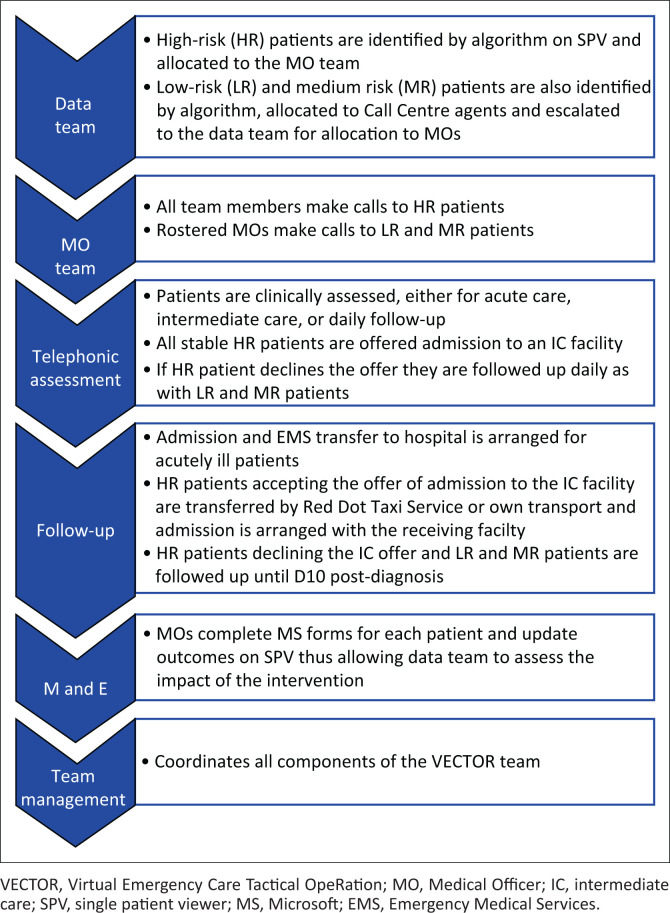
Virtual Emergency Care Tactical OpeRation team workflow.

Team management provides a structural framework and coordinates communication between the various role-players and components. A virtual communication strategy is effectively maintained by means of WhatsApp groups, emails and weekly MS Teams meetings. A workflow summary of the VECTOR team is depicted in Figure 2.

## Qualitative outcomes

Much of the positive feedback we have received on the VECTOR programme is a qualitative reflection of the patient experience. The project has emphasised patient-centred communication and the development of virtual doctor–patient relationships, and the responses that both the VECTOR team and the WCDoH have received from the public clearly depict these components. In addition, the MOs making the calls have found this work to be emotionally rewarding and sustainable. This augurs well for the delivery, uptake and acceptability of these services in the future.

## Quantitative outcomes

Beyond the rich qualitative aspects referred to, the VECTOR programme could demonstrate a notably measurable impact. Preliminary outcome measures were descriptively reported by comparing the period just before the intervention, factoring in a 2-week period to exclude any partial intervention or data lags, with the period immediately following initiation (Table 1). As the intervention was phased in over two months, the various cohorts had differing analytic periods.

**TABLE 1:** Preliminary descriptive outcome measures per risk strata.

Variable	Pre-intervention baseline mortality (2 weeks prior to launch)	Post-intervention mortality (launch to Nov 2020)	Relative change in mortality
**High-risk COVID-19 diabetic cases**			
Total high-risk cases	765	1359	-
Total high-risk deaths	221	310	-
Mortality (%)	28.8	22.8	−20.8
**Moderate-risk COVID-19 diabetic cases**			
Total moderate-risk cases	571	670	-
Total moderate-risk deaths	52	45	-
Mortality (%)	9.1	6.7	−26.4
**Low-risk COVID-19 diabetic cases**			
Total low-risk cases	215	445	-
Total low-risk deaths	21	20	-
Mortality (%)	9.8	4.5	−54.1

Note: These are preliminary outcome measures prior to the second wave, do not account for confounding and should be interpreted with caution. A formal review to account for these factors will be carried out to validate or refute the descriptive findings.

COVID-19, coronavirus disease 2019.

Accounting for the differing analytic periods, the team saw reductions in mortality across all risk strata in excess of 20%. These data still need to be formally validated and reviewed to account for possible confounders and biases but demonstrate early signs of a potentially significant impact. This has also been achieved in a cost-effective manner at a total cost-to-company of approximately R728 000.00 prior to the second wave averaging out at R19.00 per call for the first 3842 patients each receiving an average of 10 calls. To date, by the end of March 2021, the team had managed 10 928 patients (4935 high-risk patients, 45.1%; 4148 moderate-risk patients, 38%, and 1845 low-risk patients – 16.9%).

## Conclusion

This article describes how a telehealth service grew organically in the grips of the first COVID-19 wave, using high quality data and other existing capacity to drive meaningful change. The service is data-driven, patient-centric and has high levels of ownership amongst the leadership of the WCDoH. Discussions are already underway to use the telehealth platform for other conditions that lend themselves to this approach in a post COVID-19 era. The dots appear to be connected. Pressure in the system has been alleviated and patients are demonstrably benefiting.
